# Diagnostic utility of saline infusion doppler sonohysterography in endometrial mass lesions

**DOI:** 10.12669/pjms.322.9452

**Published:** 2016

**Authors:** Bilge Ogutcuoglu, Cihan Karadag, Cihan Inan, Zehra Nihal Dolgun, Ahmet Tevfik Yoldemir, Lale Aslanova

**Affiliations:** 1 Dr. Bilge Ogutcuoglu, Medeniyet University Goztepe Training and Research Hospital, Department of Obstetrics and Gynecology, Istanbul, Turkey; 2 Dr. Cihan Karadag, Marmara University Pendik Training and Research Hospital, Department of Obstetrics and Gynecology, Istanbul, Turkey; 3 Dr. Cihan Inan, Department of Obstetrics and Gynecology, Trakya University, Edirne, Turkey; 4 Dr. Zehra Nihal Dolgun, Department of Obstetrics and Gynecology, Trakya University, Edirne, Turkey; 5 Dr. Ahmet Tevfik Yoldemir, Marmara University Pendik Training and Research Hospital, Department of Obstetrics and Gynecology, Istanbul, Turkey; 6 Dr. Lale Aslanova, Marmara University Pendik Training and Research Hospital, Department of Obstetrics and Gynecology, Istanbul, Turkey

**Keywords:** Saline infusion doppler sonohysterography, Hysteroscopy, Endometrial Mass Lesions

## Abstract

**Objective:**

To evaluate the importance of saline infusion Doppler sonohysterography (SIS-D) in the assessment of transvaginal ultrasound (TVUSG)-suspected intrauterine mass lesions in women complaining about abnormal uterine bleeding with respect to hysteroscopy (H/S) and pathologic diagnosis.

**Methods:**

This study was conducted on patients, who visited to our clinic with abnormal uterine bleeding and whose TVUSGs indicated intrauterine masses. The study covered a total of 100 patients. SIS-D and hysteroscopy were performed on those 100 patients. SIS-D results were compared with hysteroscopy results. The relation between SIS-D findings and pathology results were evaluated.

**Results:**

For SIS; specificity was 96%, sensitivity was 60%, positive predictive value (PPV) was 87.8%, negative predictive value (NPV) was 83.3%, and the accuracy rate was 87%. For TVUSG; PPV was 75%. According to SIS-D, 92.2% of the lesions that had single-vessel feeding patterns were endometrial polyps, and this was statistically significant (p<0.0001). 57.1% of the lesions that had multiple-vessel feeding patterns were submucous myomas, and this was statistically significant (p<0.0001).

**Conclusion:**

SIS should be performed in patients before hysteroscopy because it will protect a considerable number of patients from unnecessary invasive procedures. SIS-D gives an idea on the histopathology of the mass.

## INTRODUCTION

Abnormal uterine bleeding is an important gynecological problem frequently experienced by women. In such cases, the cause might be a simple dysfunctional uterine bleeding or an underlying severe organic pathology. The most common organic causes for abnormal uterine bleeding include endometrial polyps, uterus leiomyomas, endometrial hyperplasia and endometrium cancer. The incidence of endometrial polyps in abnormal uterine bleeding varies around 10-30%.^1^,^2^ Uterus leiomyomas are the most common pelvic tumors in women. Submucous myomas are adjacent to endometrium, and they grow towards the endometrium and form a bulging in endometrial cavity.

The most commonly used models for abnormal uterine bleeding evaluation are TVUSG, SIS, H/S, endometrial biopsy, dilation and curettage (D&C).^3^ The first option for evaluating abnormal uterine bleeding is TVUSG.^4^ The main limitation with TVUSG is the high rate of false negativity in detecting focal intrauterine pathology. In that case, advanced methods such as SIS and H/S are needed to evaluate and view the uterine cavity. H/S is the gold standard for detecting the endometrial polyps, submucous myomas and intrauterine synechia in the uterine cavity.^5^ It also provides an opportunity for a simultaneous treatment.

The purpose of this study was to examine the diagnostic value of SIS-D in patients who came with abnormal uterine bleeding and whose TVUSGs indicated a suspected intrauterine mass. The success of SIS-D in indicating intrauterine lesions was assessed by comparing the H/S results with pathological results of the masses excised during H/S and the materials obtained by probe curettage performed afterwards.

## METHODS

This study covered a total of 100 patients including 85 premenopausal patients and 15 postmenopausal patients, who visited to the Gynecology Clinic of Istanbul Medeniyet University Goztepe Training and Research Hospital between May 2011 and May 2012 with abnormal uterine bleeding and whose TVUSGs indicated suspected masses in endometrium. Approval was received from the ethics council of Goztepe Training and Research Hospital. Informed consent forms were received from all patients. Demographical information of all patients, menstruation cycles, bleeding patterns (amount, spotting, off-cycle bleeding) systemic diseases, previous operations, drugs used (hormonal therapy) and obstetric backgrounds were asked and noted. The patients with coagulation disorders, pregnancy or suspected pregnancy, and vaginal or cervical pathology that might cause bleeding were not included in the study. All procedures (ultrasonography, SIS and H/S) were performed by two gynecologists together.

When the patient first visited, TVUSG (Sono Scape S11, Shaanxi Aipu Medical Instrument, China) was performed independently from the cycle day. 4.0-8.0 MHz vaginal probe (Sono Scape 6V1) was used. During TVUSG, cervix, endometrium, myometrium and ovaries were evaluated. SIS was performed before the 10^th^ day in the follicular period after the first menstruation in the premenopausal patients who had an image of a mass or suspected mass with clear or unclear limits in endometrium, and SIS was performed in postmenopausal patients in the earliest period without active vaginal bleeding.

The patient was put into dorso-lithotomy position for SIS. Speculum was inserted. Vulva vagina and cervix were cleaned with 10% iodized solutions. The 8f foley catheter was used for SIS. The catheter was placed in the cavity and the balloon was inflated. Speculum was removed carefully. TVUSG probe was inserted to the vagina. 20cc saline solution (sterile, 0.9%) was slowly introduced through the cannula to the cavity by using a 50cc injector until enough distention was achieved, and meanwhile uterine cavity was evaluated in longitudinal and transvers planes. Data regarding the sizes and locations of lesions, whether there was a blood feeding in the longitudinal section (by doppler), and if so, whether it was single-vessel or multiple-vessel were detected, and noted in the patient data form. H/S was planned for those patients afterwards.

Hysteroscopy (Karl Storz, Germany) was performed on all patients after the end of menstruation in the first 10 days of the follicular period of the cycle. For hysteroscopy, oblique telescope with advanced view and 4-mm diameter was used. The patients were put into dorso-lithotomy position under general anesthesia. Speculum was inserted. Vaginal and cervical cleaning was performed with 10% iodized solutions. Cervix was grasped with a single-toothed tenaculum, and cervical dilatation was performed up to Hegar dilatator number 9. Fluids including 5% mannitol were used to separate the uterus walls and ensure distention with a pressure of 80-120 mmHg through Histeromat (Storz, Germany). Cervical channel was evaluated while the hysteroscope was passing the cervical os. Uterine cavity was examined in detail. The existing end.polyps or submucous myomas (if any) were excised with the help of electro-cautery by using 80 watt cut. Probe curettage was performed on all patients after H/S with cannulas of 6--8 mm diameter (Karman, Medbar, Turkey). All materials obtained were sent to pathology. All lesions seen during H/S, the excisions performed and complications (if any) were noted in the H/S record form.

The data were recorded in the SPSS v.22.0 Windows database (IBM Corp., SPSS Statistics for Windows, Version 22.0. Armonk, NY: IBM Corp.) for statistical analysis. Pearson Chi-Square test was used to evaluate the relation between independent categorical variables. Significance level was p<0.05.

## RESULTS

The study included a total of 100 patients in the age range of 23-61 (42.05±8.92) including 85 premenopausal and 15 postmenopausal patients. The lesions, lesion sizes, locations detected during the procedures performed on patients, and blood feeding of lesions via Doppler during SIS along with the number of vessels (single, multiple), if any are indicated in Table-I.

**Table-I:** TVUSG, SIS and H/S results.

	TVUSG	SIS-D	H/S
Lesion; n(%)	100(100%)	82(82%)	75(75%)
Lesion size (mm) Min-Max	5-44	5-40	5-40
Mean±SD	17.42±7.96	18.25±7.58	18.57±7.96
*Lesion location*			
Anterior	44(44%)	39(47.6%)	36(48%)
Posterior	28(28%)	25(30.5%)	22(29.3%)
Fundal	26(26%)	10(12.2%)	9(12%)
Right lateral	1(1%)	5(6.1%)	5(6.7%)
Left lateral	1(1%)	3(3.6%)	3(4%)
Blood feeding in Doppler	88(88%)	78(95.1%)	
Single vessel		64(78%)	
Multiple vessel		14(17.1%)	

According to the pathology results of the lesions excised during H/S and endometrial samples taken from patients; 63 of 100 patients had endometrial polyps, 11 patients had submucous myomas, 10 patients had endometrial hyperplasia, one patient had a malignity developing on endometrial polyp basis, and 15 patients had endometrium in proliferative phase. Since malignity case develops on an endometrial polyp base, it was considered as a lesion that can form a mass in cavity in the study.

Table-II indicates the results where SIS is evaluated by taking H/S, the gold standard, as basis. While TVUSG diagnosed 100 patients with intrauterine masses, H/S found pathologies that might cause intrauterine masses in 75 of them. Thus, the positive predictive value of TVUSG was found to be 75%.

**Table-II:** Diagnostic performance of SIS.

Specificity	96%
Sensitivity	60%
PPV	87.8%
NPV	83.3%
Accuracy rate	87%

For the 63 cases whose pathology results were endometrial polyps, H/S found masses in endometrial cavity in 63 cases (100%), and SIS found them in 60 cases (95,2%). For the 11 cases whose pathology results were submucous myomas, both H/S and SIS found masses in endometrial cavity in all cases. For the one case whose pathology result was malignancy, both H/S and SIS found the mass in endometrial cavity since malignancy had developed on an endometrial polyp base. Table-III indicates the pathology results of the lesions detected in SIS according to blood feeding in doppler.

**Table-III:** Feeding patterns in SIS-D and biopsy pathology comparison.

			Endometrial Polyp	Submucous myoma	Endometrial hyperplasia	Cancer on endometrial polyp base	Normal mucosa	Total
Blood feeding type	Single	n(%)	59(92.2%)*	3(4.7%)	2(3.1%)	0(0%)	0(0%)	64(100.0%)
	Multiple	n(%)	1(7.1%)	8(57.1%)*	3(21.4%)	1(7.1%)	1(7.1%)	14(100.0%)
	None	n(%)	3(13.6%)	0(0%)	5(22.7%)	0(0%)	14(63.6%)*	22(100.0%)

Total		n(%)	63(63.0%)	11(11.0%)	10(10.0%)	1(1.0%)	15(15.0%)	100(100.0%)

Normal Mucosa: Endometrium in Proliferative Phase

* (p<0.01)

## DISCUSSION

Endometrial polyps are the most common cause of abnormal uterine bleeding in patients with a suspected mass in uterine cavity. In the study by Gumus et al. on postmenopausal patients who had suspected lesions in uterine cavity according to TVUSG, H/S found endometrial polyps in 51.9%.^6^ In our study, endometrial polyps were found in 63% of our patients, and this was in compliance with the literature. The literature reports that the incidence of submucous myomas was 6-10% in patients who had abnormal uterine bleeding due to a mass in uterine cavity.^7^ In our study, according to final pathology reports, the incidence of submucous myomas was 11%. Our result was also in compliance with the literature.

In our study, PPV of TVUSG was 75%, the sensitivity of SIS was 96%, its specificity was 60%, PPV was 87.8%, and NPV was 83.3%. Grimbizis et al. conducted a study on 105 patients and reported that, compared to SIS and TVUSG, H/S had a significantly higher accuracy rate in detecting intra-cavitary lesions and that SIS had a higher accuracy rate compared to TVUSG.^8^ In a recent study by Chawla et al., the sensitivity of SIS in intra-cavitary lesions was 89.1%, and its specificity was 100%, PPV was 100%, and NPV was 73.7%.^9^ When we compare our study with the results in literature, we see that specificity was low. In our study, SIS had a considerably high accuracy rate in indicating the existing lesions and finding their locations. However, the low specificity is considered to be an important disadvantage because it can increase the unnecessary H/S cases. We can explain the main cause of low specificity with the fact that, in our study 10 cases results were endometrial hyperplasia. In cases of endometrial hyperplasia, the irregularities in cavity indicate a suspected polypoid lesions in SIS, and they were considered as masses in cavity. This error caused the specificity to be lower than expected.

In our study, both SIS and H/S indicated masses in cavity in all patients whose pathology results were submucous myomas. For 63 patients whose pathology results were endometrial polyps, H/S indicated masses in cavity in all of them (100%), while SIS indicated masses in cavity in 60 of them (95.2%). The study by Luterek et al. found that SIS and H/S had similar success rates in detecting submucous myomas, and that H/S was better at detecting end.polyps.^10^ In a study that was conducted by Erdem et al. and evaluated 133 patients, the sensitivity of SIS in detecting end.polyps was 100% and its specificity was 91.8%.^11^ Bingol et al. reported that the sensitivity and negative predictive value of SIS was 100% for endometrial polyps; while its sensitivity was 99% and positive predictive value was 96% for submucousal myomas. The same study found that the sensitivity of H/S in indicating pathologies in whole uterine cavity was 98%, its specificity was 83%, PPV was 96%, and NPV was 91%. It concluded that SIS was more valuable than TVUSG and that H/S was the gold standard.^12^

In the present study, H/S indicated a mass in cavity in all patients whose pathology results were endometrial polyps and submucous myomas. H/S indicated no mass in cavity in patients whose pathology results were endometrial hyperplasia and endometrium proliferative phase. Soguktas et al. conducted a study on 89 premenopausal patients with abnormal uterine bleeding and found polypoid lesion in most cases, and reported that H/S was the method with the highest rate of accuracy. They found that, for polypoid lesions, the accuracy rate was 89% for SIS and 77% for TVUSG.^13^ In our study, the accuracy rate of SIS was 87%, and this was in compliance with the literature.

Performing a doppler during SIS can increase the accuracy of diagnosis and might give an idea on the histopathology of the existing mass. We found that 92.2% of lesions fed by a single vessel were endometrial polyps, and 57.1% of lesions fed by multiple vessels were submucous myomas. This finding can be interpreted as the success of doppler performed during SIS in determining lesion type. When we look in the literate about Doppler we can see that there are studies with transvaginal ulstrasonography and these studies show that submucous myomas are fed by multiple vessels, endometrial polyps are fed by a single vessel.^14^-^16^ We think that doppler should be performed during SIS, so that we can increase our accuracy and have an idea about histopathology. In our study, doppler performed during SIS did not show a feeding pattern in the majority of patients whose pathology results were normal, and this also indicates the importance and necessity of doppler here.

## CONCLUSION

When compared to hysteroscopy, SIS-D is an important tool in the differential diagnosis of endometrial mass lesions with its rapid and reliable determination on the histopathology of the mass with minimal invasive intervention. When used accurately, it will protect a considerable number of patients from unnecessary invasive procedures.
